# H5N1 influenza virus-specific miRNA-like small RNA increases cytokine production and mouse mortality via targeting poly(rC)-binding protein 2

**DOI:** 10.1038/cr.2018.3

**Published:** 2018-01-12

**Authors:** Xihan Li, Zheng Fu, Hongwei Liang, Yanbo Wang, Xian Qi, Meng Ding, Xinlei Sun, Zhen Zhou, Ying Huang, Hongwei Gu, Limin Li, Xi Chen, Donghai Li, Quan Zhao, Fenyong Liu, Hua Wang, Jin Wang, Ke Zen, Chen-Yu Zhang

**Affiliations:** 1 State Key Laboratory of Pharmaceutical Biotechnology, Jiangsu Engineering Research Center for MicroRNA Biology and Biotechnology, Nanjing Advanced Institute for Life Sciences (NAILS), School of Life Sciences, Nanjing University, Nanjing, Jiangsu 210046, China; 2 Central Laboratory, Nanjing Integrated Traditional Chinese and Western Medicine Hospital, Affiliated with Nanjing University of Chinese Medicine, Nanjing, Jiangsu 210014, China; 3 Jiangsu Provincial Center for Disease Control and Prevention, Nanjing, Jiangsu 210009, China; 4 Nanjing Drum Tower Hospital, Medical School of Nanjing University, Nanjing, Jiangsu 210023, China; 5 School of Public Health, University of California at Berkeley, Berkeley, CA 94720, USA

**Keywords:** H5N1, microRNA, cytokine

## Abstract

Infection of H5N1 influenza virus causes the highest mortality among all influenza viruses. The mechanisms underlying such high viral pathogenicity are incompletely understood. Here, we report that the H5N1 influenza virus encodes a microRNA-like small RNA, miR-HA-3p, which is processed from a stem loop-containing viral RNA precursor by Argonaute 2, and plays a role in enhancing cytokine production during H5N1 infection. Mechanistic study shows that miR-HA-3p targets poly(rC)-binding protein 2 (PCBP2) and suppresses its expression. Consistent with PCBP2 being an important negative regulator of RIG-I/MAVS-mediated antiviral innate immunity, suppression of PCBP2 expression by miR-HA-3p promotes cytokine production in human macrophages and mice infected with H5N1 virus. We conclude that miR-HA-3p is the first identified influenza virus-encoded microRNA-like functional RNA fragment and a novel virulence factor contributing to H5N1-induced 'cytokine storm' and mortality.

## Introduction

The sporadic transmission of the highly pathogenic H5N1 avian influenza virus from poultry to human beings in many countries highlights the threat to public health. Since 2003, over 600 laboratory-confirmed H5N1 infections have been reported in 15 nations with the mortality rate exceeding 50%^1^. Human diseases due to H5N1 virus infection can be characterized by multiple organ disorder, neurological disease and viral pneumonia with acute respiratory distress syndrome (ARDS)^2^. Among all the diseases caused by H5N1 virus infection, ARDS is the main reason of H5N1 human mortality. One widely accepted pathogenesis model for ARDS is that H5N1 infection causes dysregulation of the host innate immune response, leading to an undue discharge of chemokines and proinflammatory cytokines^3,4,5^. The 'cytokine storm' results in a great influx of inflammatory cells into respiratory tract, thereby causing 'collateral' damage to the lung tissue and inhibiting oxygen change in the alveoli^6^. The H5N1 virus replication, however, is not suppressed by this excessive release of chemokines and proinflammatory cytokines^7^, thus the continuous viral replication and increasing proinflammatory cytokine release form a vicious cycle. Therefore, finding a way to effectively control the 'cytokine storm' associated with H5N1 infection is essential for patient survival. Previous reports have shown that viral proteins (such as NS1 and PB1-F2) could affect the virulence of highly pathogenic H5N1 viruses through modulating the host cell innate immune responses^8,9,10,11^. However, the mechanisms that govern the H5N1-mediated release of proinflammatory cytokines and chemokines remain unclear.

MicroRNAs (miRNAs) belong to the category of single-stranded small (19-25 nucleotides) RNAs that can inhibit the expression of specific mRNAs via binding to complementary sequences within the target mRNAs^12,13,14^. To date, over 250 viral miRNAs have been identified, and almost all of them are encoded by DNA viruses or retroviral RNA viruses^15,16,17,18^. Thus, the lack of viral miRNAs encoded by RNA virus has led many to infer that normal viruses with RNA genomes are not amenable to the exploitation of miRNA pathway because processing of the hairpin would result in the degradation of the viral RNA genome and mRNAs^17^. However, different from most RNA viruses that transcribe and replicate their genome in the cytoplasm of infected cells, influenza A viruses complete these processes in the nucleus of the infected cells^19^. In addition, the genome of influenza A viruses consists of eight negative-sense, single-stranded RNA segments (vRNA), which serve as templates for transcription of viral mRNAs and complementary RNAs (cRNAs)^19^. The vRNAs and cRNAs might be able to avoid cleavage because they can form ribonucleoprotein complexes (vRNPs) via associating with the virus polymerase and nucleoprotein (NP) monomer^19^. Single-stranded mRNA molecules can also protect themselves from degradation by forming secondary structures via interactions through complementary segments^20^. Recently, miRNA-like small RNAs have been reported to be produced by RNA viruses such as West Nile virus and Dengue virus^21,22^. All of these prompted us to hypothesize that the H5N1 influenza virus, as an RNA virus, may encode miRNA-like small RNA fragments, which in turn, can contribute to the deadly H5N1 infections in a similar way as certain exogenous miRNAs reported in modulating the host cell functions^23,24^.

In the present study, employing Solexa sequencing, northern blot and quantitative RT-PCR assays, we confirmed that H5N1 influenza virus encodes a miRNA-like small RNA, miR-HA-3p. Mechanistic study shows that miR-HA-3p contributes to the 'cytokine storm' during H5N1 infection via targeting the poly(rC)-binding protein 2 (PCBP2), an important negative regulator of RIG-I/MAVS-mediated antiviral innate immunity. The results derived from both virus-infected human primary macrophage and mouse model suggest that miR-HA-3p serves as an important virulence factor contributing to H5N1-induced macrophage inflammatory responses and mouse mortality.

## Results

### H5N1 influenza virus encodes a miRNA-like small RNA, miR-HA-3p

**Figure 1:**
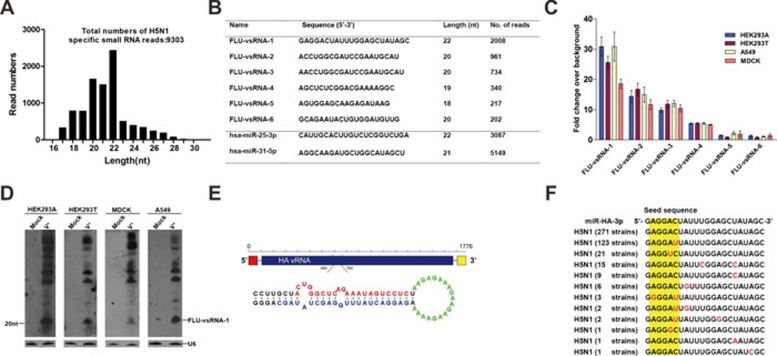
Identification of microRNA-like small RNA fragments encoded by H5N1 influenza virus. **(A)** Size distribution of H5N1-derived small RNAs in H5N1 virus-infected A549 cells (MOI = 1) detected by Solexa sequencing. **(B)** Sequences and reads of H5N1-derived small RNAs in H5N1-infected A549 cells detected by Solexa sequencing. **(C)** Levels of six H5N1-encoded small RNAs in H5N1-infected cells detected by quantitative RT-PCR (qRT-PCR). All values were relative to that of mock-infected cells and normalized to U6. **(D)** Northern blot analysis of total RNAs isolated from H5N1- or Mock-infected cells. DIG-labeled LNA probe complementary to the sequence of FLU-vsRNA-1 was used. The cellular small RNA U6 was also probed on each blot as a loading control. **(E)** Genomic positions and predicted stem-loop secondary structure of H5N1 encoded pre-miR-HA-3p. Red: 5′ sequence; green: loop sequence; blue: 3′ sequence. **(F)** The conservation of predicted miR-HA-3p in 455 highly pathogenic H5N1 strains.

To determine whether H5N1 influenza virus is capable of encoding miRNA-like small RNA, small RNA (< 30 nt) extracted from A549 cells infected with H5N1 influenza virus (A/Jiangsu/1/2007(H5N1), MOI = 1) for 48 h was sequenced by Solexa deep-sequencing technology. The results showed that H5N1-infected A549 cells contain a panel of H5N1-derived small RNA fragments (GEO accession number: GSE67222). In total, 9 303 reads of 466 different H5N1-derived small RNAs were obtained from 10 374 143 total reads (Figure 1A). Six H5N1-derived small RNAs displayed more than 200 reads (Figure 1B). Among these H5N1-derived small RNAs, FLU-vsRNA-1 had the highest copy number, which was in a similar range of functional endogenous miR-25-3p and miR-31-5p (Figure 1B). To further confirm the existence of H5N1-encoded small RNAs, a stem-loop reverse transcription followed by quantitative RT-PCR (qRT-PCR) was performed for all six small RNAs (Figure 1C) using RNA derived from HEK293A, HEK293T, A549 and MDCK cells infected with A/Jiangsu/1/2007(H5N1) (MOI = 1) for 48 h. The expression levels of viral small RNAs were normalized to levels of U6 snRNA, and values were presented relative to RNA derived from mock-infected cells. Four small RNAs with relative higher read number were readily detected in all cells at 48 h post-infection, while the other two viral small RNAs were expressed at very low levels (Figure 1C). We further confirmed the expression of FLU-vsRNA-1 in total RNAs isolated from HEK293A, HEK293T, MDCK and A549 cells infected with A/Jiangsu/1/2007(H5N1) by northern blot analysis (Figure 1D).

To determine whether H5N1-encoded FLU-vsRNA-1 is a miRNA-like small RNA fragment, we developed a computer algorithm, based on structural features and resemblance to known miRNA precursors, to screen for the typical hairpin-like stem-loop structure of miRNA precursors. The secondary structures of candidate miRNAs were predicted using the Mfold algorithm^25^. After applying the algorithm to the reference genome sequences obtained from the National Center for Biotechnology Information (NCBI) database, we predicted one precursor of FLU-vsRNA-1 at the 3p arm and thus re-named FLU-vsRNA-1 as miR-HA-3p (Figure 1E) given that the predicted precursor is derived from the coding region of viral HA segment. Because of the high mutation rate of the influenza virus genome, we examined the evolutionary conservation of the viral-derived miR-HA-3p by aligning the miR-HA-3p sequence with currently published full-length HA sequences of 455 highly pathogenic H5N1 strains, and found that the predicted miRNA was highly conserved among 455 H5N1 strains (Figure 1F). Thus, miR-HA-3p was not limited to a particular isolate but was highly conserved among highly pathogenic H5N1 influenza viruses.

### Biogenesis of miR-HA-3p is dependent on the unique hairpin structure of viral pre-miRNA and Ago2

**Figure 2:**
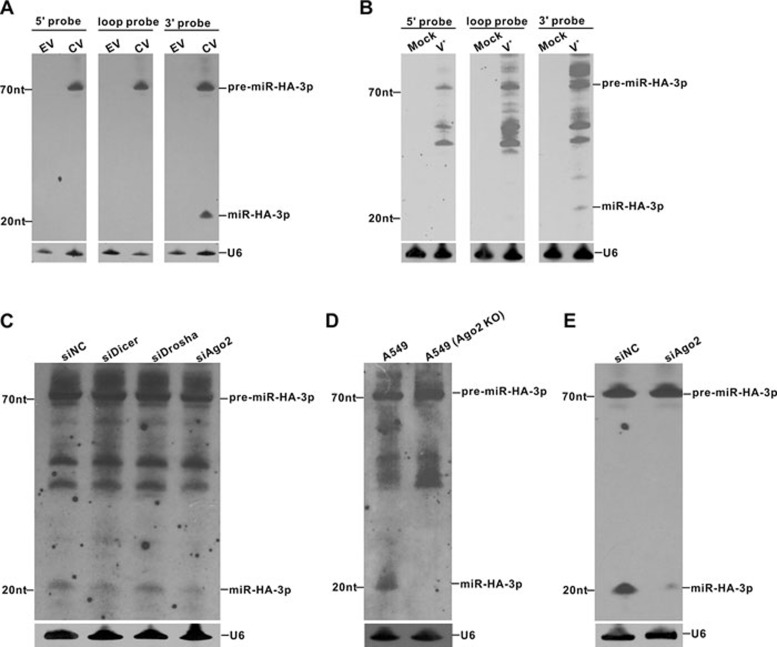
Biogenesis of miR-HA-3p is dependent on the hairpin structure of viral pre-miRNA and Ago2. **(A)** Northern blot analysis of total RNAs isolated from A549 cells transfected with pcDNA6.2-pre-miR-HA-3p or empty vector at 48 h post-transfection. DIG-labeled LNA 5′ probe, loop probe and 3′ probe, which are complementary to the sequence derived from the 5′ stem, the terminal loop and the 3′ stem of the pre-miRNA were used. **(B)** Northern blot analysis of miR-HA-3p in H5N1- or mock-infected A549 cells with three different probes mentioned above at 48 h post-infection. **(C)** Northern blot analysis of total RNAs isolated form A549 cells transfected with Dicer siRNA (siDicer), Drosha siRNA (siDrosha), Ago2 siRNA (siAgo2) or control siRNA (siNC) before H5N1 virus infection. DIG-labeled LNA probe complementary to the sequence of miR-HA-3p was used. **(D)** Northern blot analysis of miR-HA-3p level in A549 cells and Ago2-knockout A549 cells infected with H5N1 virus at 48 h post-infection. DIG-labeled LNA probe complementary to the sequence of miR-HA-3p was used. **(E)** Northern blot analysis of total RNAs isolated from A549 cells transfected with siAgo2 or siNC before transfected with pcDNA6.2-pre-miR-HA-3p. DIG-labeled LNA probe complementary to the sequence of miR-HA-3p was used. EV, empty vector; CV, coding vector.

To further demonstrate that miR-HA-3p is generated from the specific viral pre-miRNA but not a random degradation product of the viral genome, we cloned the pre-miR-HA-3p sequence into the vector pcDNA6.2, transfected the resulting plasmid into A549 cells and analyzed cellular RNAs by northern blot with the three specific locked nucleic acid (LNA)-modified oligonucleotide probes: a 5′ probe, a 3′ probe and a loop probe, which are complementary to the sequence derived from the 5′ stem, the 3′ stem and the terminal loop of the pre-miRNA, respectively (Figure 2A). As shown in Figure 2A, a 22-nt miR-HA-3p was clearly detected using the 3′ probe, confirming that miR-HA-3p is indeed produced from the specific pre-miRNA sequence and not a random degradation product from the viral genome. To further investigate whether the miRNAs detected by Solexa and qRT-PCR are indeed produced in H5N1-infected cells, A549 cells were infected with A/Jiangsu/1/2007(H5N1). Total RNAs isolated from the infected cells were subjected to northern blot analysis with three different probes mentioned above. The miR-HA-3p of 22 nt, as well as the presumably pre-miR-HA-3p of ∼70 nt, were clearly detected using the 3′ probe, while only larger RNAs but no 22-nt RNA were detected using either the 5′ probe or the loop probe (Figure 2B). The fact that this 22-nt small RNA has a discrete sequence composition strongly suggests that it is not a degradation product from larger RNAs but miRNA processed from the specific pre-miRNA. In addition, A549 cells infected with another H5N1 influenza virus isolate, A/Anhui/2/2005, also produced miR-HA-3p (Supplementary information, Figure S1), confirming that generation of miR-HA-3p in the host-infected cells is a common phenomenon for various H5N1 influenza virus isolates. Next, we tested whether the generation of viral miR-HA-3p shares the biogenesis pathway of mammalian miRNA. Given that Dicer and Drosha play key roles in the biogenesis of miRNAs^26,27,28^ and Ago2 is also involved in the maturation of several atypical miRNAs through cleaving pre-miRNAs^29^, we knocked down Dicer, Drosha or Ago2 in A549 cells, respectively, using RNA interference (RNAi) approach. The cells with or without knockdown of Dicer, Drosha or Ago2 were then infected with A/Jiangsu/1/2007(H5N1). Knockdown efficiency of cellular Dicer, Drosha or Ago2 was confirmed by western blot analysis (Supplementary information, Figure S2A-S2C). We found that there was a significant decline in miR-HA-3p production in Ago2-silenced cells compared with cells transfected with control siRNA, whereas no difference was observed in Dicer-knockdown or Drosha-knockdown cells (Figure 2C). To confirm that Ago2 plays a key role in the biogenesis of miR-HA-3p, we further knocked out Ago2 in A549 cells using CRISPR-Cas9 technique^30^ and then infected the cells with A/Jiangsu/1/2007(H5N1). Knockout of Ago2 was confirmed by western blot analysis (Supplementary information, Figure S2D). Subsequently, the levels of miR-HA-3p were analyzed using northern blot. The result showed no mature miR-HA-3p was detected in Ago2-knockout cells, whereas the signal of pre-miR-HA-3p was increased (Figure 2D). This result further suggests the role of Ago2 in the biogenesis of the miR-HA-3p. The same results were obtained when Ago2 was silenced in A549 cells before they were transfected with pcDNA6.2-pre-miR-HA-3p (Figure 2E). Taken together, these results indicate that maturation of viral miR-HA-3p follows non-classical, Ago2-dependent but Dicer and Drosha-independent pathway.

### MiR-HA-3p targets host cell poly(rC)-binding protein 2

**Figure 3:**
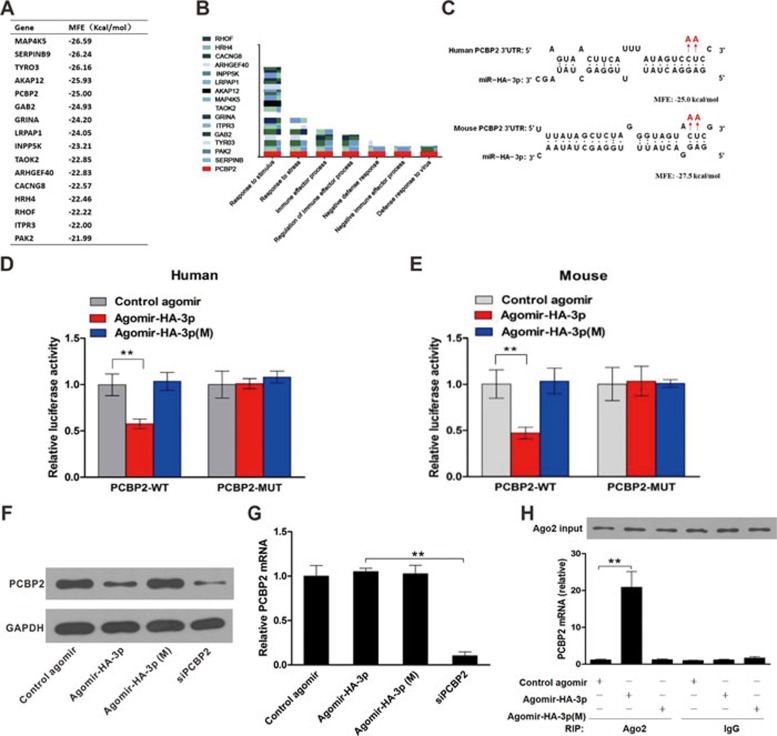
MiR-HA-3p reduces PCBP2 expression via targeting the 3′ UTR of PCBP2 mRNA. **(A)** The list of possible target genes of miR-HA-3p analyzed using the Targetscan and RNAhybrid algorithms. **(B)** The predicted target genes of miR-HA-3p classified by the GO databases based on biological process. **(C)** The predicted binding site for miR-HA-3p in PCBP2 3′ UTR. In 3′ UTR mutant, replaced nucleotide (red) was indicated by the arrows. **(D, E)** Luciferase activity in HEK293T cells transfected with plasmid encoding wild-type (WT) or mutated (MUT) 3′ UTR of human **(D)** or mouse **(E)** PCBP2 plus control agomir, agomir-HA-3p or mutant agomir-HA-3p. **(F, G)** Levels of PCBP2 protein **(F)** and mRNA **(G)** in A549 cells transfected with control agomir, agomir-HA-3p, mutant agomir-HA-3p or PCBP2 siRNA. The expression of GAPDH was analyzed as a control. **(H)** PCBP2 mRNA level in the immunoprecipitated complexes from A549 cells transfected with control agomir, agomir-HA-3p or mutant agomir-HA-3p using anti-Ago2 antibody or control IgG; Top, western blot of Ago2 protein in A549 cells (input control). Data are presented as the mean ± SEM (*n* = 3). ^**^P < 0.01.

Influenza virus is generally regarded to be recognized by the host innate immune system through three major pattern recognition receptors (PRRs), the Toll-like receptors, the retinoic acid-inducible gene I (RIG-I-) and the NOD-like receptor family^31^. These PRRs and their respective downstream signaling play an essential role in stimulating the expression of pro-inflammatory cytokines and type I interferons. To explore the function of miR-HA-3p in modulating H5N1 infection-induced cytokine storm, we performed target gene analysis using the Targetscan and RNAhybrid algorithms^32,33^. MiR-HA-3p has total 16 possible target genes in human genome (Figure 3A). Using the Gene ontology (GO) analysis^34^, we clustered the identified genes into groups based on their biological processes. These clusters included proteins that are involved in negative regulation of defense response (GO: 0031348), regulation of immune effector process (GO: 0002697), regulation of defense response to virus (GO: 0050688), regulation of response to stimulus (GO: 0048583), negative regulation of immune effector process (GO: 0002698), regulation of response to stress (GO: 0080134) and immune effector process (GO: 0002252) (Figure 3B). Given that identified genes are mainly involved in the regulatory pathway of host cell defense response to virus, we thus focused on the differentially expressed proteins within these pathways.

As PCBP2, a member of the hnRNP E family of RNA binding proteins that interact with a sequence-specific motif of single-stranded poly(C) tracts, is involved in both regulation of defense response to virus and negative regulation of immune effector process, we selectively tested whether PCBP2 served as a target gene of miR-HA-3p in H5N1-induced uncontrolled immune reaction. In addition to regulating mRNA translation and maintaining mRNA stability^35^, PCBP2 is essential for the prevention of excessive immune responses by serving as a pivotal negative regulator in the MAVS signaling pathway^36^. MAVS-mediated antiviral signaling has been well recognized as a major mechanism for host cell defense against infection by viruses, particularly H5N1^37,38^. As a negative regulator, PCBP2 can interact with MAVS, leading to its degradation^36^. Therefore, excessive expression or knockdown of PCBP2 would suppress or promote the cellular inflammatory response to viral infection, respectively. In this case, H5N1-encoded miR-HA-3p may enhance MAVS-mediated antiviral signaling by silencing its negative regulator PCBP2, leading to an excessive immune response or the cytokine storm during H5N1 infection. The predicted interaction between miR-HA-3p and the target sites in the PCBP2 was illustrated in Figure 3C. The minimum-free energy value of the hybridization was −25 kcal/mol, and the value was well within the range of genuine miRNA-target pairs. Furthermore, target analysis for miR-HA-3p was also performed in mouse, and similar results were obtained. To test whether H5N1-derived miR-HA-3p regulates PCBP2 expression, the entire PCBP2 3′-UTRs of the human or mouse were inserted into expression vector, immediately next to the firefly luciferase open-reading frame. In addition, we generated constructs with two nucleotide mutations in the 'seeding' sequence of the 3′-UTR of PCBP2 (Figure 3C). As shown in Figure 3D and 3E, we observed that miR-HA-3p agonist (agomir-HA-3p) significantly downregulated the expression of firefly luciferase fused to the PCBP2 wild-type 3′-UTR, while mutated miR-HA-3p agonist (agomir-HA-3p(M)) did not. In the reciprocal experiment, in which HEK293T cells were transfected with the firefly luciferase vector fused with the mutated PCBP2 3′-UTR, neither agomir-HA-3p nor agomir-HA-3p(M) had an effect on the luciferase activity (Figure 3D and 3E). These results demonstrated that miR-HA-3p is able to directly target the sequences in the 3′-UTR of PCBP2 mRNA.

To test whether miR-HA-3p is sufficient to downregulate PCBP2 protein expression, we examined the protein level of PCBP2 in the presence of miR-HA-3p in A549 cells. As a control, an RNAi construct specific for PCBP2 that can substantially diminish PCBP2 expression was employed. After transfection of agomir-HA-3p into A549 cells, the PCBP2 expression was significantly inhibited as determined by western blot analysis, but agomir-HA-3p(M) could not affect the protein expression of PCBP2 (Figure 3F). The ability of miR-HA-3p to downregulate PCBP2 protein expression was as effective as that of siPCBP2. In contrast, no difference in PCBP2 mRNA transcript level was observed between cells treated with agomir-HA-3p and control agomir (Figure 3G), suggesting that miR-HA-3p plays a role in translational repression but not mRNA degradation. To gain direct evidence for the targeting of PCBP2 by miR-HA-3p, an RNA-binding protein immunoprecipitation experiment was performed to detect the association of PCBP2 mRNA with Ago2, a crucial component of functional RNA-induced silencing complex (RISC). As shown in Figure 3H, in A549 cells transfected with agomir-HA-3p, there was an ∼20-fold enrichment for Ago2-bound PCBP2 mRNA, suggesting that miR-HA-3p physically interacts with PCBP2 mRNA in the RISC and thus regulates PCBP2 protein level post-transcriptionally. As expected, there was no enrichment for Ago2-bound PCBP2 mRNA when agomir-HA-3p(M) was expressed (Figure 3H).

### MiR-HA-3p enhances proinflammatory cytokine production in human macrophages infected with H5N1

**Figure 4:**
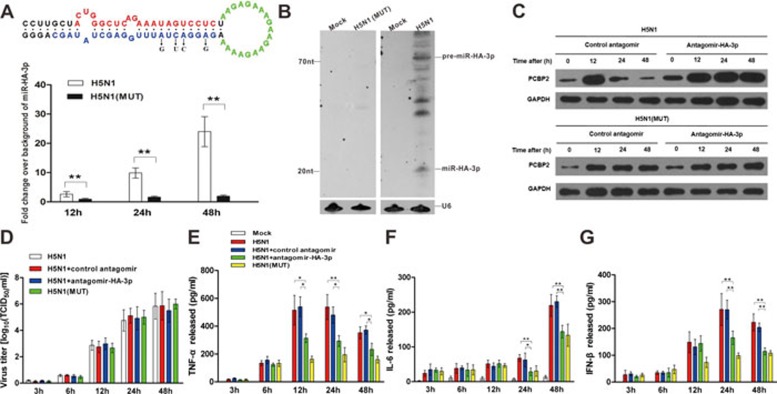
Inhibition of miR-HA-3p reduces cytokine production in primary macrophages during H5N1 virus infection. **(A)** Levels of miR-HA-3p in primary macrophages infected with H5N1 or mutant H5N1 virus at different time points. Up: Schematic description of mutation site of H5N1 mutant. Down: Fold change of miR-HA-3p levels in primary macrophages detected by quantitative RT-PCR (qRT-PCR). **(B)** Northern blot analysis of miR-HA-3p using total RNAs extracted from primary macrophages infected with H5N1 or mutant H5N1 virus at 48 h post-infection. DIG-labeled LNA probe complementary to the sequence of miR-HA-3p was used. **(C)** PCBP2 protein levels in primary macrophages electroporated with control antagomir or miR-HA-3p antagomir prior to infection with H5N1 or mutant H5N1 viruses at different time points. **(D)** Viral titers of H5N1 or mutant H5N1 viruses in virus-infected primary macrophages determined by TCID_50_ assay using MDCK cells. **(E-G)** Levels of TNF-α **(E)**, IL-6 **(F)** and IFN-β **(G)** in culture supernatants of macrophages infected with H5N1 or mutant H5N1 viruses plus different treatments. Data are presented as the mean ± SEM (*n* = 3). ^*^P < 0.05. ^**^P < 0.01.

In general, the negative-sense ssRNA genome of the H5N1 virus can be recognized by RIG-I via its adaptor protein MAVS^38^. RIG-I/MAVS then activates the downstream signaling molecules to produce proinflammatory cytokines and type I interferons^6^. Given that H5N1-infected patients generally have significantly higher levels of cytokines and chemokines compared with those with seasonal influenza virus infection, we postulated that the H5N1-derived miR-HA-3p might play a role in promoting cytokine and chemokine production during H5N1 infection via regulation of the RIG-I signaling pathway. As PCBP2 is the key negative regulator in RIG-I/MAVS-mediated signaling, miR-HA-3p might modulate RIG-I/MAVS-mediated signaling via targeting PCBP2. Macrophages are a major source of cytokine production during H5N1 influenza virus infection^2^, thus human monocyte-derived macrophages (MDMs) were used to study the effect of miR-HA-3p on cytokine production. In the experiment, we electroporated human MDMs with control agomir, agomir-HA-3p or mutant agomir-HA-3p(M), respectively. At 24 h post-transfection, intracellular miR-HA-3p levels were significantly upregulated by agomir-HA-3p treatment, whereas miR-HA-3p levels in MDMs treated with control agomir or agomir-HA-3p(M) remained unchanged (Supplementary information, Figure S3A). The PCBP2 protein levels were markedly downregulated by agomir-HA-3p compared to treatment with control agomir or agomir-HA-3p(M) (Supplementary information, Figure S3B), while the mRNA levels of PCBP2 were not significantly affected by these treatments (Supplementary information, Figure S3C). To determine the specificity of miR-HA-3p's effect on the expression of target proteins in the context of viral infection, we performed reverse genetics to generate an A/Jiangsu/1/2007(H5N1) mutant virus with four mutations of the 'seed' nucleotides (nucleotides 2, 5, 6 and 8 at the 5′ end of the mature miRNA) without altering the amino acid sequence (Figure 4A). To assess the effect of the mutation on viral replication *in vitro*, we infected macrophages with viruses at MOI of two. Supernatants of the infected cells were harvested at 3, 6, 12, 24 and 48 h post-infection and subjected to virus titration. No substantial differences were observed in the titers of infectious wild-type and mutant viruses (Supplementary information, Figure S4). We then detected the expression pattern of miR-HA-3p in H5N1 influenza virus-infected MDMs. As shown in Figure 4A, successful disruption of miR-HA-3p expression was confirmed by qRT-PCR analysis. MDMs infected with the wild-type H5N1 influenza virus began to express relatively high levels of miR-HA-3p 24 h post-infection, whereas the levels of miR-HA-3p remained low in MDMs infected with mutated H5N1 virus. The disruption of miR-HA-3p expression was further confirmed by northern blot analysis (Figure 4B).

To test the role of miR-HA-3p in regulating H5N1-induced cytokine secretion, we electroporated MDMs with miR-HA-3p antagonist (antagomir-HA-3p) or control antagomir prior to the virus infection. As shown in Figure 4C, the protein levels of PCBP2 were significantly upregulated by antagomir-HA-3p compared to treatment with control antagomir at 24 and 48 h post-infection. As expected, antagomir-HA-3p had no effect on the PCBP2 protein levels when infected with mutant H5N1 virus. To exclude the possibility of the off-target effects of electroporation on virus replication, the titers of infectious virus in untreated MDMs or those treated with antagomir-HA-3p or control antagomir were assessed by titration in MDCK cells. No difference in the titers of infectious virus was detected (Figure 4D). To rule out the side effect of the control antagomir, we examined the effect of various antagomirs. For this experiment, three antagomirs targeting two randomly selected RNA sequences at upstream and downstream of miR-HA-3, and one sequence of miR-HA-3p with four bases mutated, were synthesized. Macrophages were transfected with antagomir-HA-3p or various control antagomirs via electroporation, followed by H5N1 infection and virus titer detection at 3, 6, 12, 24 and 48 h post-infection. As shown in Supplementary information Figure S5, control antagomir-1, neither control antagomir-2, antagomir-HA-3p (MUT) nor antagomir-HA-3p affected the virus titers in H5N1-infected macrophages compared with 'empty' electroporation (buffer only), suggesting that control antagomirs and antagomir-HA-3p have no effect on viral replication. The mRNA level of TNF-α, a well-documented cytokine associated with H5N1 influenza virus infection, was measured at various time points after infection. Different from the control antagomir, MDMs treated with antagomir-HA-3p displayed a downregulated transcription of TNF-α at 12, 24 and 48 h post-infection (Supplementary information, Figure S6). As IL-6 is part of the cytokine cascade triggered by TNF-α, IL-6 mRNA was also quantified. At 24 h post-infection, the level of IL-6 mRNA in MDMs treated with antagomir-HA-3p was only 60% of that detected in cells treated with the control antagomir (Supplementary information, Figure S6). We observed similar results for other cytokines, including interferon β (IFN-β) and interleukin 1β (IL-1β) (Supplementary information, Figure S6). At the same time points, cytokine concentrations were measured in the supernatants of infected macrophages using ELISA. Supernatants from cultured macrophages treated with antagomir-HA-3p had lower concentrations of TNF-α, IL-6 and IFN-β than those treated with the control antagomir at 24 and 48 h post-infection (Figure 4E-4G). In fact, the levels of TNF-α, IL-6 and IFN-β in the supernatants from antagomir-HA-3p-treated MDMs were similar to those from MDMs infected with the mutant H5N1 virus. Moreover, we have also assessed the levels of TNF-α and IL-6 secreted by H5N1-infected macrophages at various time points following the treatment with control antagomir-1, control antagomir-2, antagomir-HA-3p (MUT) or antagomir-HA-3p. The results showed that only antagomir-HA-3p but not control antagomirs reduced the levels of TNF-α and IL-6 in macrophages (Supplementary information, Figure S7).

### MiR-HA-3p contributes to the 'cytokine storm' and mouse mortality induced by H5N1 infection

**Figure 5:**
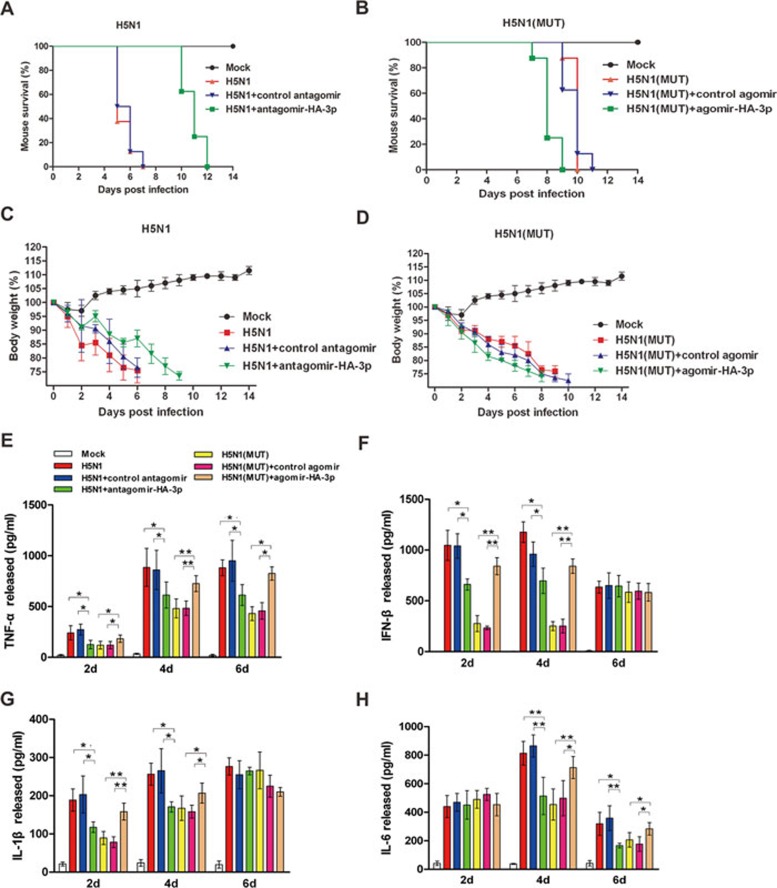
Inhibition of miR-HA-3p increased mouse resistance to H5N1 virus infection. **(A)** Survival rate of mice with different treatments following intranasal inoculation with 10^3^ EID_50_ of H5N1 virus (*n* = 8). **(B)** Survival rate of mice with different treatments following intranasal inoculation with 10^3^ EID_50_ of mutant H5N1 virus (*n* = 8). **(C)** Body weight of mice with different treatments after inoculation with 10^3^ EID_50_ of H5N1 virus (*n* = 8). **(D)** Body weight of mice with different treatments after inoculation with 10^3^ EID_50_ of mutant H5N1 virus (*n* = 8). **(E-H)** Levels of TNF-α **(E)**, IFN-β **(F)**, IL-1β **(G)** and IL-6 **(H)** in mouse lungs following intranasal inoculations with H5N1 or mutant H5N1 viruses plus different treatments. Data are presented as the mean ± SEM (*n* = 3). ^*^P < 0.05. ^**^P < 0.01.

**Figure 6:**
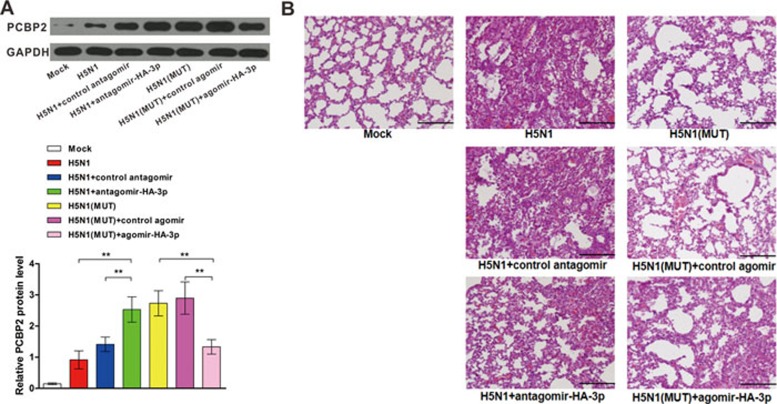
PCBP2 protein levels and histopathological effect on mouse lung tissues following H5N1 virus infection and different treatments. **(A)** Upper panel: representative western blot image of PCBP2 protein levels in mouse lungs on day 4 post-infection with H5N1 virus. **(A)** Lower panel: the quantitative analysis of the PCBP2 protein level in upper panel. Data are presented as the mean ± SEM (*n* = 3). ^**^P < 0.01. **(B)** Representative images of histopathological effect of H5N1 infection on mouse lungs. Formalin-fixed, paraffin-embedded lung tissue sections from mice on day 4 post-infection were stained with hematoxylin and eosin. Note that lungs from H5N1-infected mice and H5N1-infected mice treated with control antagomir display acute neutrophil infiltration and necrosis of the bronchial and bronchiolar epithelium with considerable sloughing and disruption of the bronchial lining epithelium. In contrast, lungs from H5N1 virus-infected mice treated with antagomir-HA-3p or mutant H5N1 virus-infected mice are less severely damaged and display a mild to moderate neutrophil infiltration across the bronchial epithelium. Scale bars, 500 μm.

To investigate the role of miR-HA-3p in H5N1-induced cytokine dysregulation *in vivo*, we used an established murine model of H5N1 infection. Groups of female BALB/c mice (6-week-old) were inoculated intranasally with a lethal dose (10^3^ EID_50_) of H5N1 or mutated H5N1 viruses, and morbidity (measured by weight loss), mortality, virus replication and cytokine concentrations were determined. After 8 h of viral infection, mice were intravenously injected with 25 mg/kg of saline-formulated antagomir-HA-3p or control antagomir for 5 consecutive days. As shown in Figure 5, inoculation of mice with H5N1 resulted in 100% lethality (Figure 5A) and > 20% loss of bodyweight on day 7 after inoculation (Figure 5C). Control antagomir had no effect on H5N1-induced mouse lethality and bodyweight loss. However, when H5N1-infected mice were treated with antagomir-HA-3p, all mice survived on day 9 post-inoculation though the mice still showed a considerable bodyweight loss. These results suggest that depletion of miR-HA-3p in H5N1-infected mice by antagomir-HA-3p can significantly decrease the mouse mortality. This conclusion of miR-HA-3p as a fatal virulence factor for H5N1 was also supported by our experiment using mutated H5N1 virus. As expected, mice inoculated with H5N1(MUT) showed a longer survival (Figure 5B) and a slower bodyweight loss (Figure 5D) compared to H5N1-infected mice (Figure 5A and 5C). However, when H5N1(MUT)-infected mice were further treated with agomir-HA-3p, mouse survival rate (Figure 5B) was markedly decreased and mouse bodyweight loss was increased (Figure 5D). As expected, treatment with control agomir had no effect on mouse survival rate and bodyweight loss induced by H5N1(MUT) infection. To determine whether the production of cytokines during H5N1 influenza virus infection was affected by miR-HA-3p, the concentrations of TNF-α, IFN-β, IL-1β and IL-6 in mouse lungs from all groups were measured at different time points after infection. A comparison between the groups revealed that H5N1-infected mice showed higher levels of TNF-α, IFN-β, IL-1β and IL-6 than did H5N1(MUT)-infected mice or mice infected with H5N1 but treated with antagomir-HA-3p (Figure 5E-5H). We further compared the virus titers in mouse lung homogenates after inoculation with H5N1 or H5N1(MUT) and found no substantial differences between the virus titers in H5N1-infected mice and H5N1(MUT)-infected mice (Supplementary information, Figure S8). However, on day 4 post-infection, lungs of mice infected with H5N1 virus had a significantly lower level of PCBP2 protein compared to that of mice infected with H5N1 virus but at the same time treated with antagomir-HA-3p or mice infected with mutant H5N1 virus (Figure 6A). Elevated of miR-HA-3p level was also detected in lung tissues and serum from mice inoculated with H5N1 but not H5N1(MUT) (Supplementary information, Figure S9). Histological examination revealed that depleting miR-HA-3p by antagomir-HA-3p protected mouse lungs from inflammatory damage, as shown in Figure 6B. There was significant and extensive inflammation and necrosis of the bronchial and bronchiolar epithelium that was often accompanied by a sloughing of the epithelium in the lungs of H5N1-infected mice. In contrast, the lungs of mice inoculated with H5N1 but treated with antagomir-HA-3p showed a moderate focal inflammation in the large airways on day 4 post-infection, a condition that was similar to the lungs of mice infected with H5N1(MUT). Therefore, removal of the miR-HA-3p resulted in a virus that caused mild inflammation that was confined to the large airways in the lungs and less severe lung pathology than caused by the wild-type H5N1 virus. These observations were consistent with the results derived from the primary macrophages, indicating that miR-HA-3p promoted cytokine production during H5N1 infection by suppressing PCBP2.

## Discussion

In the present study, we have identified a miRNA-like small RNA unique for H5N1 virus, miR-HA-3p. Through reducing PCBP2 expression in the infected host cells such as macrophages, miR-HA-3p inhibits the negative regulation of the RIG-I/MAVS pathway, leading to an exacerbation of cytokine production and inflammatory response of host cells. To our knowledge, this is the first finding on the influenza virus-encoded miRNA-like small RNA and its biological function during viral infection.

Our results demonstrated that upon virus infection, the biogenesis of miR-HA-3p in the host cells followed an atypical miRNA biogenesis pathway. Although miR-HA-3p is derived from the coding region of viral HA segment, its maturation is independent of Dicer and Drosha. Northern blot analysis of various pre-miR-HA-3p mutants indicated that the biogenesis of miR-HA-3p is dependent on the unique hairpin structure of pre-miR-HA-3p. Like Dengue virus-encoded miRNA-like DENV-vsRNA-5 recently reported by Mazhar and Sassan^22^, the maturation process from pre-miR-HA-3p to miR-HA-3p in the host cells is not due to RNA random degradation but is dependent on Ago2. The biogenesis of miR-HA-3p in the infected host cells reveals a novel pathway for producing miRNA: with a unique hairpin structure, a pre-miRNA can be generated from viral genome by an unknown mechanism; once a pre-miRNA is produced, it can be rapidly cleaved by host Ago2 to form mature miRNA.

Structural analysis and functional study further identified miR-HA-3p as a miRNA-like small RNA. First, Solexa sequencing and northern blot using various probes indicated that miR-HA-3p is an intact miRNA. In H5N1-infected A549 cells, both miR-HA-3p of 22-nt and pre-miR-HA-3p of ∼70-nt were detected using the 3′ probe, while no 22-nt RNA but larger RNAs were detected using either the 5′ probe or the loop probe (Figure 2B), strongly arguing that miR-HA-3p is not a degradation product from larger RNAs but a miRNA product processed from the specific pre-miRNA. Second, like a typical miRNA, multiple potential targets have been predicted for miR-HA-3p. One of these targets, PCBP2, is selected because it has been shown to be involved in both regulation of host defense response to virus and negative regulation of host immune effector process. Specifically, PCBP2 can interact with MAVS, leading to proteasomal degradation of MAVS^36^, thus functioning as an important negative regulator of RIG-I/MAVS-mediated antiviral innate immunity^31^. Bioinformatics analysis and experimental validation have confirmed PCBP2 as a conserved target of H5N1-encoded miR-HA-3p and reduction of PCBP2 by miR-HA-3p plays a key role in cytokine overproduction in H5N1-infected cells. Finally, miR-HA-3p executes its function in a traditional miRNA manner. As shown in Figure 3H, miR-HA-3p is directly associated with Ago2, indicating that miR-HA-3p targets PCBP2 through its association with Ago2, a crucial component of functional RNA-induced silencing complex. In addition, although miR-HA-3p is as effective as PCBP2-specific siRNA (siPCBP2) in downregulating PCBP2 protein expression, it plays a role in translational repression but not mRNA degradation (Figure 3G).

Given the unprecedented severity of H5N1 disease and its continued threat to public health, H5N1 infection has been extensively studied and various factors are found to be involved in the pathogenesis of H5N1 influenza^2^. Here, we report a viral miRNA-like small RNA, miR-HA-3p, as a new player in modulating host immune responses induced by H5N1 infection. Different from other influenza viruses such as H1N1 and H3N2, H5N1 contains unique sequences that can form a hairpin structure and is able to be processed to pre-miR-HA-3p and miR-HA-3p, consequently. Genomic analysis shows that miR-HA-3p is relatively conserved, suggesting that production of miR-HA-3p is a common mechanism which plays an essential role in cytokine storm and mortality induced by various highly pathogenic H5N1 subtypes. Moreover, mice treated with antagomir-HA-3p show a considerable resistance to H5N1 infection with significantly less weight loss and longer survival time. As miR-HA-3p is conserved across various H5N1 strains, miR-HA-3p may serve as a key factor in promoting excessive cytokine production in various H5N1 infections. More importantly, antagomir-HA-3p treatment would be an effective strategy to attenuate and control the overboard inflammatory responses induced by infection with various highly pathogenic H5N1 strains. In summary, our study demonstrates for the first time that the highly pathogenic H5N1 influenza virus can encode a viral miRNA-like small RNA, miR-HA-3p, which plays a critical role in promoting the 'cytokine storm' during H5N1 infection via suppressing PCBP2. This finding also provides a potentially efficient, antagomir-HA-3p-based, therapeutic strategy to treat H5N1 infection.

## Materials and Methods

### Cells, viruses and animals

HEK293A, HEK293T, Madin-Darby canine kidney (MDCK) and human alveolar epithelial (A549) cells were purchased from the China Cell Culture Center (Shanghai, China). All cell lines were maintained at 37 °C in a humidified 5% CO_2_ incubator with Dulbecco's modified eagle medium (Gibco, CA) containing 10% fetal bovine serum (FBS, Gibco), 100 units/mL of penicillin and 100 μg/mL of streptomycin. The H5N1 influenza A virus strains A/Jiangsu/1/2007(H5N1) and A/Anhui/2/2005 were obtained from Jiangsu provincial center for disease prevention and control^39^. Six-week-old, pathogen-free, female BALB/c mice were used to assay antiviral activity *in vivo*. All experiments with H5N1 virus were conducted in the enhanced animal biosafety laboratory level 3 (ABSL3) facilities.

### Ethics statement

All experiments using animals and procedures of animal care and handling were carried out in strict accordance with the recommendations in the Guide for the Care and Use of Laboratory Animals of the Ministry of Science and Technology of the People's Republic of China. The protocols for animal studies were approved by the Institutional Animal Care and Use Committee (IACUC) of Nanjing University (No. BRDW-XBS–14). The usage and handling of human blood samples in this study was approved by the Institutional Review Board of Nanjing University (No. AF/SC-07/01.0) and written informed consent was obtained from each participant.

### Solexa deep sequencing

Small RNAs (< 30 nt) were extracted from A549 cells infected with influenza virus (A/Jiangsu/1/2007(H5N1)) at an MOI of one for 48 h post-infection using the mirVana^TM^ miRNA Isolation Kit (Ambion, Austin, TX, USA). Solexa sequencing of RNA samples was performed by BGI (Shenzhen, China). After removing the adaptor sequences from the raw data, the clean reads were analyzed. All data have been uploaded to the GEO database (GEO accession number: GSE67222).

### Computational prediction of viral miRNAs

Genome sequences of H5N1 influenza A viruses were downloaded from the NCBI database (Bethesda, MD) and scanned for stretches of the typical hairpin-like stem-loop structure of miRNA precursors (pre-miRNA). The computational method used criteria that were based on the features derived from known human pre-miRNAs to discriminate miRNAs from random sequences. Then, sequence comparison was conducted for all predicted miRNA hairpins and specific stretches of miRNA hairpins that had no sequence similarity to that of human genome were selected for further experimental study.

### Prediction of viral miRNAs Targets and Gene ontology analysis

Human target genes of novel H5N1 miRNAs were predicted using TargetScan custom miRNA prediction methods^32^ and RNAhydrid^33^. GO analysis of the significant probe list was performed using PANTHER (http://www.pantherdb.org/)^34,40^, using text files containing the Gene ID list and accession numbers of the Illumina probe ID. All data analysis and visualization of differentially expressed genes were conducted using R 2.4.1 (www.r-project.org). The predicted miRNA target base-pairing schematics were produced using the online RNA folding program Mfold (http://unafold.rna.albany.edu/?q=mfold/RNA-Folding-Form).

### Quantitative RT-PCR of mature miRNA

Total RNA was extracted from cells infected with influenza virus (A/Jiangsu/1/2007(H5N1)) (MOI =1) using TRIzol Reagent according to the manufacturer's instructions (Invitrogen, Carlsbad, CA). Quantitative RT-PCR was performed using TaqMan miRNA probes (Applied Biosystems, Foster City, CA) according to the manufacturer's instructions. Briefly, total RNA was reverse transcribed to cDNA using AMV reverse transcriptase (Takara) and a stem-loop RT primer (Applied Biosystems). Quantitative PCR was performed using a TaqMan PCR kit and an Applied Biosystems 7300 Sequence Detection System (Applied Biosystems).

### Northern blot

Northern blots were performed as described previously^41,42^. Briefly, total RNA of cells infected with influenza virus (A/Jiangsu/1/2007(H5N1)) at an MOI of one for 48 h were extracted using TRIzol Reagent according to the manufacturer's instructions. Total RNA (50 μg) was separated on a 15% denaturing polyacrylamide gel. The RNA was then transferred to nylon membrane (GE healthcare) in a semi-dry Trans-Blot electrophoretic transfer cell (Bio-Rad, CA) for 1.5 h at 300 mA. DIG-labeled LNA probes (Exiqon) complementary to 5′ arm, loop or 3′ arm of the pre-miRNA were used. Hybridization and washing were performed according to standard procedures. The DIG Luminescent Detection Kit (Roche) was used for luminescent detection. The membrane was wrapped in plastic wrap and exposed to an X-ray film at room temperature.

### Generation of Ago2 Knockout A549 Cells by CRISPR/Cas9 technology

To generate Ago2-KO A549 cells, target sequences were cloned into pLentiCRISPRv2 by cutting with BsmBI as previous described^30^. The following target sequences were used: Ago2 target-1: 5′-CACCGCTCCACCTAGACCCGACTTT-3′, Ago2 target-2: 5′-CACCGAGGTCCCAAAGTCGGGTCT-3′.

### Luciferase assay

The entire human and mouse PCBP2 3′-UTR segments were amplified by PCR using human and mouse genomic DNA as a template. The PCR products were cloned into the *Spe*I and *Hind*III sites of the multiple cloning regions in pMIR-reporter plasmids (Ambion). Insertion was confirmed by sequencing. For luciferase reporter assays, 0.2 μg of firefly luciferase reporter plasmid, 0.1 μg of β-galactosidase expression vector (Ambion) and equal amounts (20 pmol) of agomir-HA-3p (a miR-HA-3p agonist) or control agomir were transfected into HEK293T cells in 24-well plates. The β-galactosidase vector was used as a transfection control. At 24 h post-transfection, cells were analyzed using a luciferase assay kit (Promega).

### Influenza virus infection of macrophages

Peripheral blood mononuclear cells (PBMCs) were isolated by Ficoll-Paque (GE healthcare) density centrifugation of whole blood from healthy donors. PBMC were plated at 2 × 10^6^ cells/mL in RPMI 1640 medium supplemented with 10% FBS in 24-well poly-𝒟-Lysine coated plates (Costar). After 24 h, non-adherent cells were removed and monocytes were allowed to differentiate into macrophages in complete medium supplemented with 100 μg/mL streptomycin, 100 U/mL penicillin and 100 ng/mL rHu GM-CSF at 37 °C under a humidified 5% CO_2_ atmosphere for 7 days. The research protocol was approved by the ethics committee of Nanjing University. Differentiated macrophages (from monocytes seeded at 3 × 10^5^ cells per well in 24-well culture plates) were infected at an MOI of two. After 30 min of virus adsorption, the virus inoculum was removed and the cells were washed with warm culture medium and incubated in macrophage SFM medium (GIBCO BRL, Gaithersburg, MD) supplemented with 50 U/mL penicillin, 50 μg/mL streptomycin and 1 μg/mL N-p-tosyl-ℒ-phenylalaninechloromethyl ketone-treated trypsin (Sigma, St Louis, MO). Samples of culture supernatant were collected for virus titration and cytokine analysis. RNA was extracted from cells for analysis of cytokine gene expression.

### Quantitative RT-PCR of mRNA

DNase-treated total RNA was extracted using TRIzol Reagent according to the manufacturer's instructions. The cDNA was synthesized from mRNA with poly (dT) primers and AMV reverse transcriptase (Takara). The cDNA samples (2 μL) were employed for quantitative PCR in a total volume of 20 μL using SYBR Green (Takara) on an Applied Biosystems 7300 Sequence Detection system. The reactions were incubated in a 96-well optical plate at 95 °C for 5 min, followed by 40 cycles of 95 °C for 15 s, 58 °C for 30 s and 72 °C for 30 s. The average of triplicate data obtained for each sample was employed to calculate the relative change in gene expression after normalization to β-actin mRNA.

### Quantification of cytokines by ELISA

Culture supernatants or mouse serum were collected and irradiated with UV light (Scienta03-αUltra Violet Cross-linker) for 15 min to inactivate infectious agents. The concentrations of cytokines were quantified by ELISA assays (R&D Systems).

### Western blot

Cells were lysed for 1 h at 4 °C with 1% (vol/vol) Nonidet P-40 in PBS with a protease-inhibitor 'cocktail', separated by SDS-PAGE and transferred onto polyvinylidenedifluoride (PVDF) membranes. The membranes were blocked for 1 h at room temperature with 5% non-fat milk in Tris-buffered saline (TBS) plus Tween 20 (TBST), followed by an overnight incubation at 4 °C with an antibody (diluted in blocking buffer) against PCBP2 (Abcam). Normalization was performed by blotting the same samples with an antibody against GAPDH.

### Immunoprecipitation

Immunoprecipitation assays were performed according to the manufacturer's instructions. Briefly, cells were washed three times with cold PBS (4 °C), scraped from each dish and then collected by centrifugation at 1 000 rpm for 5 min at 4 °C. Cells were then resuspended in an appropriate volume of complete IP lysis buffer (20 mM Tri-HCl (pH7.5), 150 mM NaCl, 0.5% Nonidet P-40, 2 mM EDTA, 0.5 mM dithiothreitol (DTT), 1 mM NaF, 1× protease inhibitor and 1× PMSF). Mouse monoclonal anti-Ago2 antibody (2 μg) was used to immunoprecipitate RNA-binding proteins. After purification, immunoprecipitated RNA was analyzed by quantitative RT-PCR for miR-HA-3p using TaqMan miRNA probes (Applied Biosystems) and for PCBP2 mRNA using specific primers or by western blot analysis using a rabbit polyclonal anti-Ago2 antibody according to the manufacturer's instructions.

### Generation of recombinant viruses by reverse genetics

All experiments were performed in approved biosafety level-3 laboratory. The H5N1 influenza virus strain A/Jiangsu/1/2007(H5N1) was grown in allantoic cavities of 10-day-old embryonated chicken eggs (Merial Vital, Beijing, China). RT-PCR was used to amplify the eight viral genes, and viral cDNAs were inserted into the dual-promoter plasmid pHW2000^43^. The plasmids were sequenced, and the QuikChange Site-Directed Mutagenesis kit (Stratagene, La Jolla, CA) was used to generate mutations in the HA plasmid. Recombinant viruses were generated by DNA transfection of MDCK/HEK293T cells. Transfection supernatant was injected into 10-day-old embryonated chicken eggs, and virus stock was prepared, sequenced and titrated.

### Virus challenge and treatments

BALB/c mice were first sedated with 2,2,2-tribromoethanol (Avertin; Sigma-Aldrich, St. Louis, MO) and intranasally infected with 10^3^ EID_50_ of wild-type or mutant viruses. At 8 h post-infection, the mice were injected intravenously with the control antagomir (designed and synthesized by Ribobio Company, two miRNAs of *C. elegans*, and bioinformatics analysis (homology comparison) suggests that these miRNAs have minimal homology compared to the genome of human, mouse and rat), antagomir-HA-3p, control agomir or agomir-HA-3p respectively (25 mg/kg body weight) for 5 days. The animals were then monitored daily for symptoms of infection, body weight and survival.

### Statistical analysis

All data from the western blot and the semi-quantitative RT-PCR analyses are representative of at least three independent experiments. Data shown are presented as the mean ± SEM of at least three independent experiments and differences are considered statistically significant at *P* < 0.05 using Student's *t*-test.

## Author Contributions

XL, HL, YW, ZF, XQ, MD, XS, YH, HG, LL and XC performed the experiments. XL and ZZ analyzed data. JW, DL and ZZ performed the computational prediction. XL, FL, KZ and CZ wrote the manuscript. XC, QZ, FY, HW, KZ and CZ designed the experiments and revised the manuscript.

## Competing Financial Interests

The authors declare no competing financial interests.

## Supplementary Information

Supplementary information, Figure S1 Northern blot analysis of miR-HA-3p using total RNA derived from A549 cells infected with H5N1 virus (A/Anhui/2/2005) at 48 h post-infection.

Supplementary information, Figure S2 Western blot analysis of protein levels of Dicer (**A**), Drosha (**B**) and Ago2 (**C**) aftertransfection of A549 cells with control or three different synthetic siRNAs at 48 h post-transfection.

Supplementary information, Figure S3 Downregulation of PCBP2 expression in primary macrophages by miR-HA-3p.

Supplementary information, Figure S4 The effect of mutations on viral replication *in vitro*.

Supplementary information, Figure S5 No effect of antagomir-HA-3p and various control antagomirs on viral replication.

Supplementary information, Figure S6 Quantitative RT-PCR assay of TNF-α, IFN-β, IL-1β or IL-6 mRNA levels in human primary macrophages infected with H5N1 or mutant H5N1 viruses plus different treatments.

Supplementary information, Figure S7 No effect of antagomir-HA-3p and various control antagomirs on cytokines secretion in H5N1-infected macrophages.

Supplementary information, Figure S8 Viral titers in mouse lungs after H5N1 or mutant H5N1 infection plus different treatments were determined by TCID_50_ assay using MDCK cells at different time points after infection.

Supplementary information, Figure S9 The levels of miR-HA-3p detected by quantitative RT-PCR in lung and serum samples of mice infected with H5N1 or mutant H5N1 viruses on day 2 and day 4 post-infection.
