# Supplementary material for: H5N1 influenza virus-specific miRNA-like small RNA increases cytokine production and mouse mortality via targeting poly(rC)-binding protein 2
Source: Cell Res. 2018 Jan 12;28(2):157–71. doi: 10.1038/cr.2018.3 (PMC5799819; doi:10.1038/cr.2018.3)
Supplement: Supplementary information, Figure S1 — Northern blot analysis of miR-HA-3p using total RNA derived from A549 cells infected with H5N1 virus (A/Anhui/2/2005) at 48 h post-infection. [file cr20183x1.pdf]

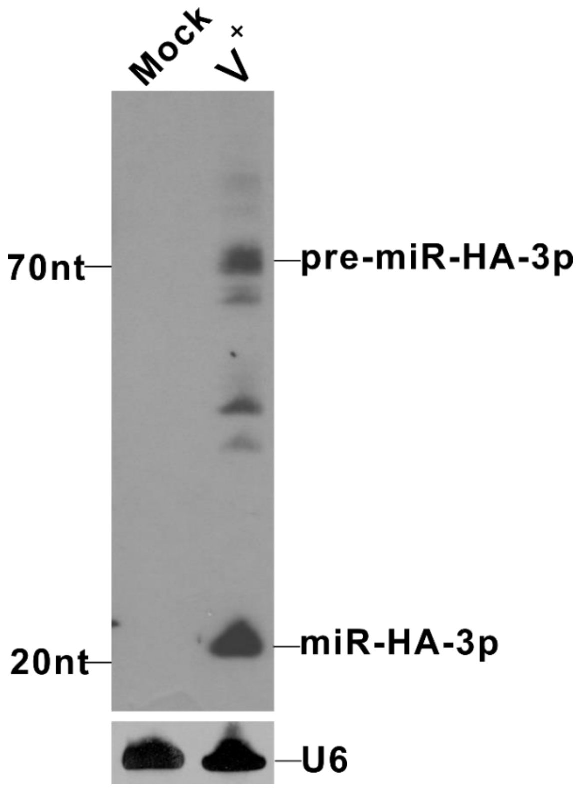

Supplementary information, Figure S1 Northern blot analysis of miR-HA-3p using total RNA derived from A549 cells infected with H5N1 virus (A/Anhui/2/2005) at 48 h post-infection.

DIG-labeled LNA probe complementary to the sequence of miR-HA-3p was used. The cellular small RNA U6 was also probed on each blot to serve as a loading control.
