# Supplementary material for: H5N1 influenza virus-specific miRNA-like small RNA increases cytokine production and mouse mortality via targeting poly(rC)-binding protein 2
Source: Cell Res. 2018 Jan 12;28(2):157–71. doi: 10.1038/cr.2018.3 (PMC5799819; doi:10.1038/cr.2018.3)
Supplement: Supplementary information, Figure S2 — Western blot analysis of protein levels of Dicer (A), Drosha (B) and Ago2 (C) aftertransfection of A549 cells with control or three different synthetic siRNAs at 48 h post-transfection. [file cr20183x2.pdf]

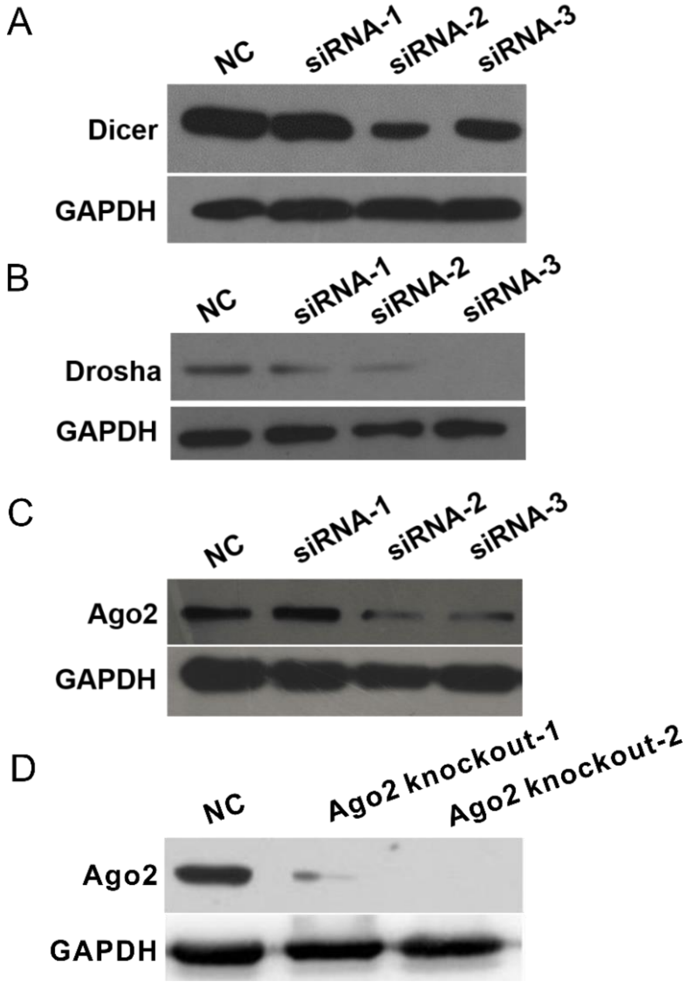

Supplementary information, Figure S2 Western blot analysis of protein levels of Dicer (**A**), Drosha (**B**) and Ago2 (**C**) after transfection of A549 cells with control or three different synthetic siRNAs at 48 h post-transfection.

The siRNA-2 (Dicer), siRNA-3 (Drosha) and siRNA-2 (Ago2) were used in the following experiments. (**D**) Western blot analysis of protein levels of Ago2 in Ago2 knockout A549 cells. The Ago2 knockout-2 cells were used in the following experiments. The expression of GAPDH was analyzed as a control.
