# Supplementary material for: H5N1 influenza virus-specific miRNA-like small RNA increases cytokine production and mouse mortality via targeting poly(rC)-binding protein 2
Source: Cell Res. 2018 Jan 12;28(2):157–71. doi: 10.1038/cr.2018.3 (PMC5799819; doi:10.1038/cr.2018.3)
Supplement: Supplementary information, Figure S3 — Downregulation of PCBP2 expression in primary macrophages by miR-HA-3p. [file cr20183x3.pdf]

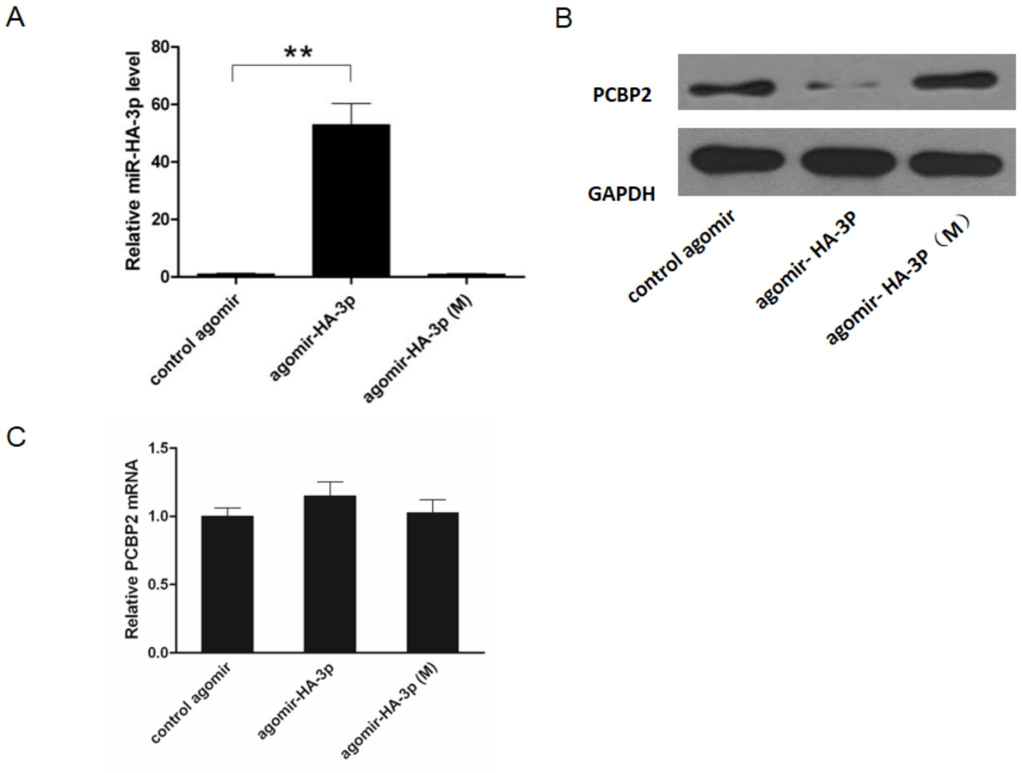

Supplementary information, Figure S3 Downregulation of PCBP2 expression in primary macrophages by miR-HA-3p.

(**A**) Relative miR-HA-3p levels in primary macrophages transfected with control agomir, agomir-HA-3p or mutant agomir-HA-3p (M) at 24 h post-transfection determined by quantitative RT-PCR. All values were normalized to levels of U6. (**B**) PCBP2 protein levels in primary macrophages transfected with control agomir, agomir-HA-3p or agomir-HA-3p (M) at 48 h post-transfection. (**C**) Quantitative RT-PCR analysis of PCBP2 mRNA levels in primary macrophages transfected with control agomir, agomir-HA-3p or agomir-HA-3p (M) at 48 h post-transfection. Data are presented as the mean ± SEM (n = 3). *, $P < 0.05$. **, $P < 0.01$.
