# Supplementary material for: H5N1 influenza virus-specific miRNA-like small RNA increases cytokine production and mouse mortality via targeting poly(rC)-binding protein 2
Source: Cell Res. 2018 Jan 12;28(2):157–71. doi: 10.1038/cr.2018.3 (PMC5799819; doi:10.1038/cr.2018.3)
Supplement: Supplementary information, Figure S4 — The effect of mutations on viral replication in vitro. [file cr20183x4.pdf]

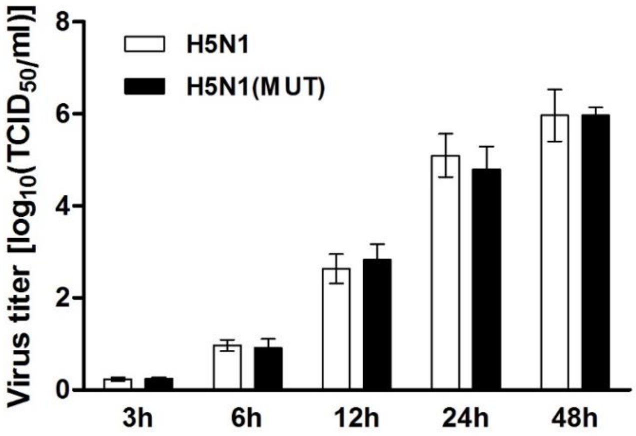

Supplementary information, Figure S4 The effect of mutations on viral replication *in vitro*.

Viral titers in the culture supernatants of primary macrophages infected with H5N1 or mutant H5N1 viruses at 3 h, 6 h, 12 h, 24 h and 48 h post-infection were determined by $TCID_{50}$ assay using MDCK cells.
