# Supplementary material for: H5N1 influenza virus-specific miRNA-like small RNA increases cytokine production and mouse mortality via targeting poly(rC)-binding protein 2
Source: Cell Res. 2018 Jan 12;28(2):157–71. doi: 10.1038/cr.2018.3 (PMC5799819; doi:10.1038/cr.2018.3)
Supplement: Supplementary information, Figure S5 — No effect of antagomir-HA-3p and various control antagomirs on viral replication. [file cr20183x5.pdf]

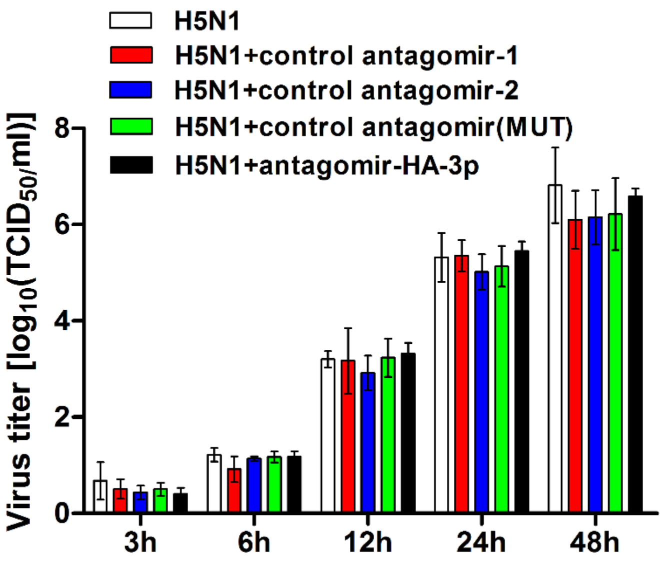

Supplementary information, Figure S5 No effect of antagomir-HA-3p and various control antagomirs on viral replication.

Viral titers in the culture supernatants of primary macrophages infected with H5N1 virus at 3 h, 6 h, 12 h, 24 h and 48 h post-infection plus different treatments were determined by $TCID_{50}$ assay using MDCK cells. Antagomir-1 target RNA sequences: AUGUGAAAUCAAACAAAUUAGU, antagomir-2 target RNA sequences: UUUAUAGAGGGAGGAUGGCAGG, antagomir-HA-3p (MUT) target RNA sequences: GGGGCUTGTTTGGAGCTATAGC.
