# Supplementary material for: H5N1 influenza virus-specific miRNA-like small RNA increases cytokine production and mouse mortality via targeting poly(rC)-binding protein 2
Source: Cell Res. 2018 Jan 12;28(2):157–71. doi: 10.1038/cr.2018.3 (PMC5799819; doi:10.1038/cr.2018.3)
Supplement: Supplementary information, Figure S6 — Quantitative RT-PCR assay of TNF-α, IFN-β, IL-1β or IL-6 mRNA levels in human primary macrophages infected with H5N1 or mutant H5N1 viruses plus different treatments. [file cr20183x6.pdf]

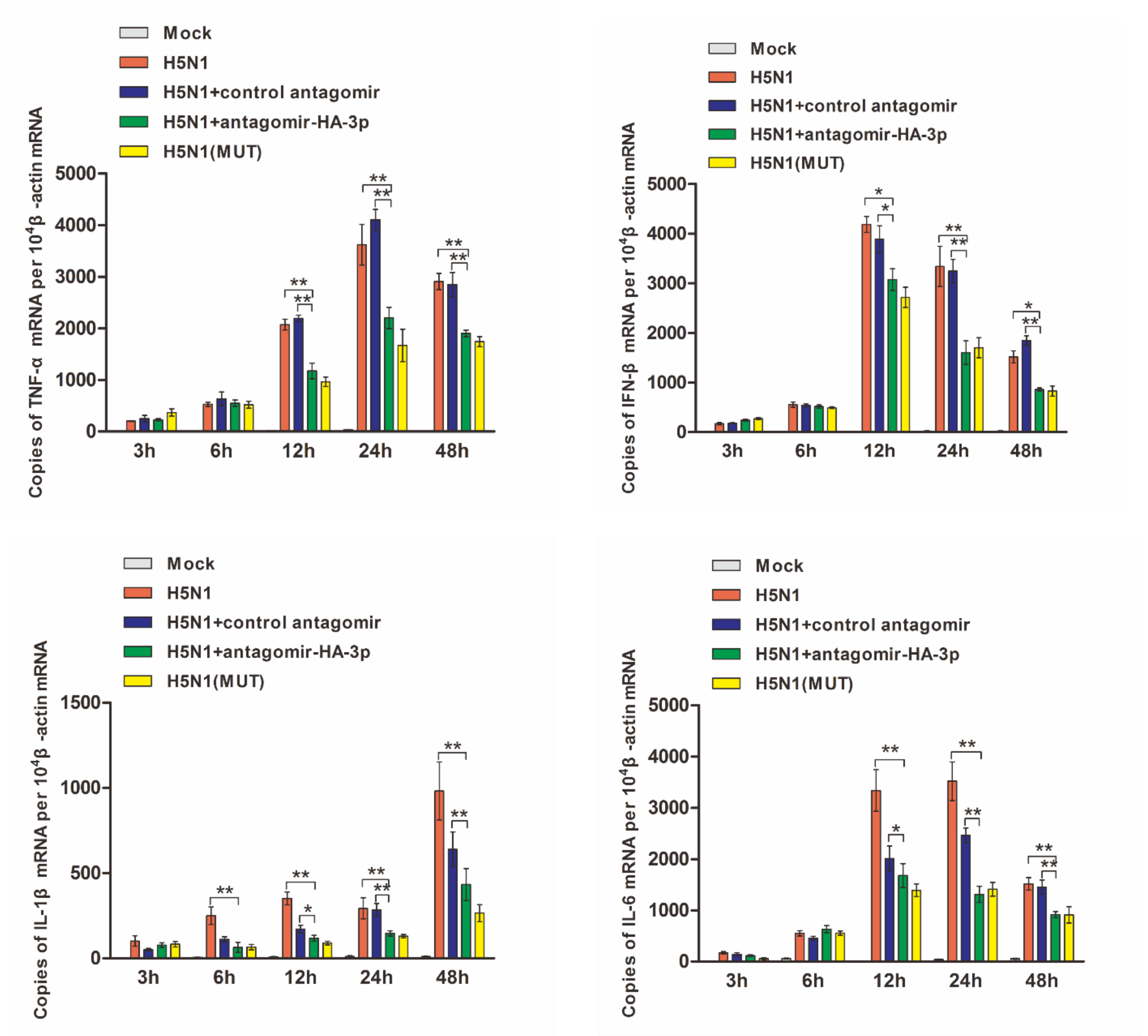

Supplementary information, Figure S6 Quantitative RT-PCR assay of TNF-α, IFN-β, IL-1β or IL-6 mRNA levels in human primary macrophages infected with H5N1 or mutant H5N1 viruses plus different treatments. Data are presented as the mean ± SEM (n = 3). The average of triplicate data obtained for each sample was used to calculate the relative change in gene expression after normalization to β-actin mRNA. *, $P < 0.05$. **, $P < 0.01$.
