# Supplementary material for: H5N1 influenza virus-specific miRNA-like small RNA increases cytokine production and mouse mortality via targeting poly(rC)-binding protein 2
Source: Cell Res. 2018 Jan 12;28(2):157–71. doi: 10.1038/cr.2018.3 (PMC5799819; doi:10.1038/cr.2018.3)
Supplement: Supplementary information, Figure S7 — No effect of antagomir-HA-3p and various control antagomirs on cytokines secretion in H5N1-infected macrophages. [file cr20183x7.pdf]

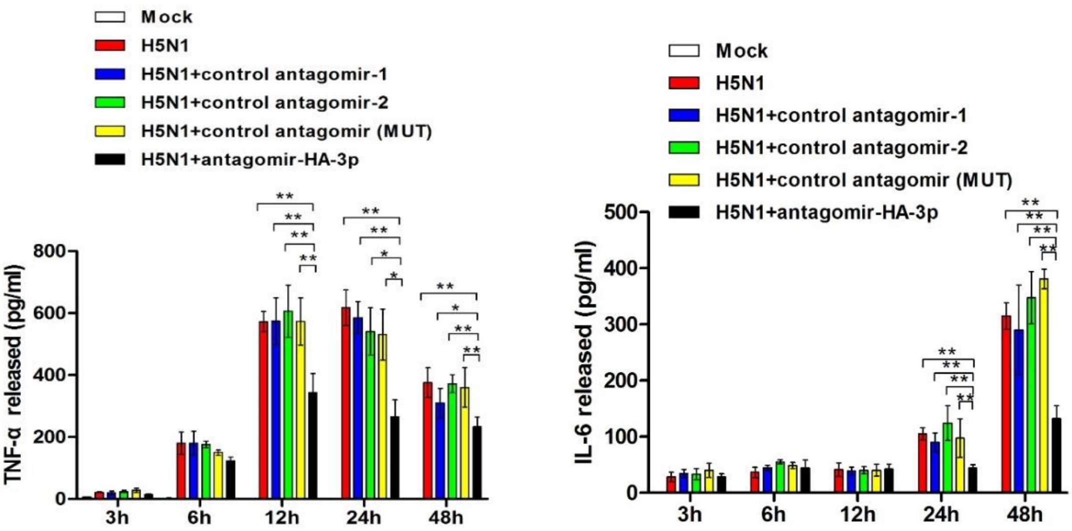

Supplementary information, Figure S7 No effect of antagomir-HA-3p and various control antagomirs on cytokines secretion in H5N1-infected macrophages.

Levels of TNF-α and IL-6 in culture supernatants of macrophages infected with H5N1 virus plus different treatments were assessed at different time points after infection. Data are presented as the mean ± SEM (n = 3). *, $P < 0.05$. **, $P < 0.01$.
