# Supplementary material for: H5N1 influenza virus-specific miRNA-like small RNA increases cytokine production and mouse mortality via targeting poly(rC)-binding protein 2
Source: Cell Res. 2018 Jan 12;28(2):157–71. doi: 10.1038/cr.2018.3 (PMC5799819; doi:10.1038/cr.2018.3)
Supplement: Supplementary information, Figure S8 — Viral titers in mouse lungs after H5N1 or mutant H5N1 infection plus different treatments were determined by TCID50 assay using MDCK cells at different time points after infection. [file cr20183x8.pdf]

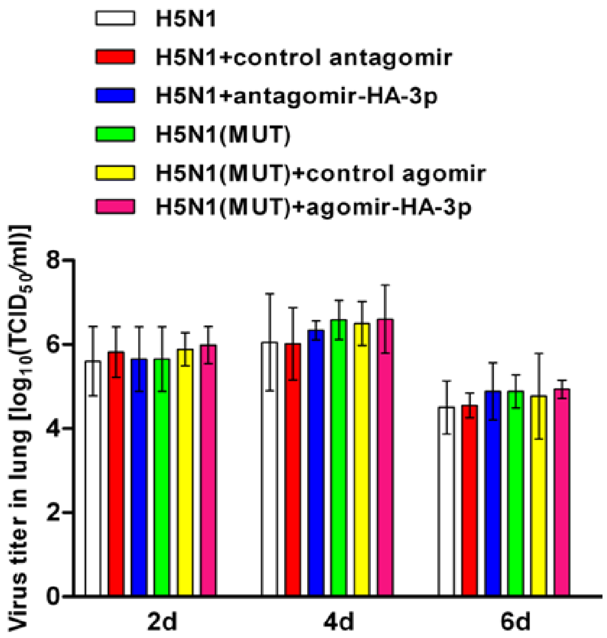

Supplementary information, Figure S8 Viral titers in mouse lungs after H5N1 or mutant H5N1 infection plus different treatments were determined by $TCID_{50}$ assay using MDCK cells at different time points after infection.
