# Supplementary material for: H5N1 influenza virus-specific miRNA-like small RNA increases cytokine production and mouse mortality via targeting poly(rC)-binding protein 2
Source: Cell Res. 2018 Jan 12;28(2):157–71. doi: 10.1038/cr.2018.3 (PMC5799819; doi:10.1038/cr.2018.3)
Supplement: Supplementary information, Figure S9 — The levels of miR-HA-3p detected by quantitative RT-PCR in lung and serum samples of mice infected with H5N1 or mutant H5N1 viruses on day 2 and day 4 post-infection. [file cr20183x9.pdf]

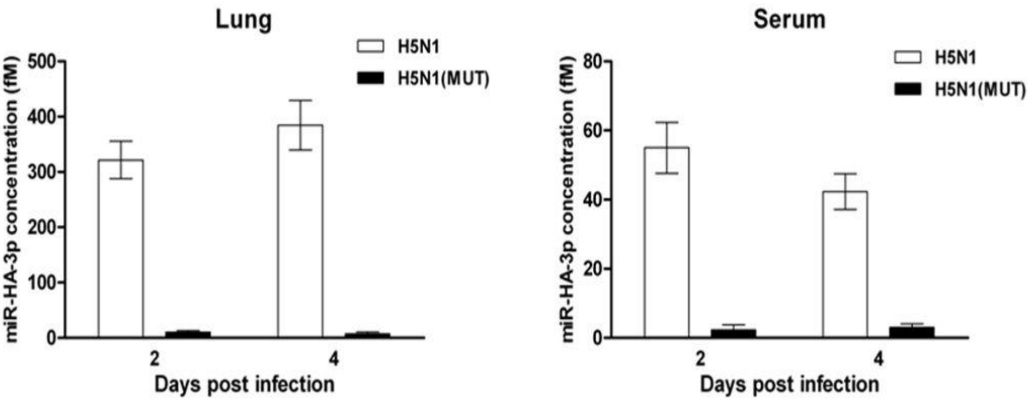

Supplementary information, Figure S9 The levels of miR-HA-3p detected by quantitative RT-PCR in lung and serum samples of mice infected with H5N1 or mutant H5N1 viruses on day 2 and day 4 post-infection. C_T values were converted to absolute values based on the standard curves.
